# A qualitative study exploring personal recovery meaning and the potential influence of clinical recovery status on this meaning 20 years after a first-episode psychosis

**DOI:** 10.1007/s00127-021-02121-w

**Published:** 2021-06-18

**Authors:** Donal O’Keeffe, Ann Sheridan, Aine Kelly, Roisin Doyle, Kevin Madigan, Elizabeth Lawlor, Mary Clarke

**Affiliations:** 1 DETECT Early Intervention in Psychosis Service, Dublin, Ireland; 2 School of Nursing and Midwifery, Trinity College Dublin, Dublin, Ireland; 3 School of Nursing, Midwifery, and Health Systems, University College Dublin, Dublin, Ireland; 4 Saint John of God Hospitaller Services, Dublin, Ireland; 5 Saint John of God Community Services, Dublin, Ireland; 6 School of Postgraduate Studies, Faculty of Medicine and Health Sciences, Royal College of Surgeons in Ireland, Dublin, Ireland; 7 School of Medicine and Medical Science, University College Dublin, Dublin, Ireland

**Keywords:** First-episode psychosis, Personal recovery, Clinical recovery, Qualitative research, iHOPE-20

## Abstract

**Purpose:**

Long-term data on recovery conceptualisation in psychotic illness are needed to support mental health services to organise themselves according to recovery-oriented frameworks. To our knowledge, no previous research has investigated how first-episode psychosis (FEP) service users (sampled across psychotic illness type) perceive recovery beyond 5 years after diagnosis. We aimed to explore personal recovery meaning with individuals 20 years after their FEP and examine the potential influence of clinical recovery status on how they defined recovery (i.e. personal recovery).

**Methods:**

Twenty participants were purposefully sampled from an epidemiologically representative FEP incidence cohort. At 20-year follow-up, semi-structured interviews were conducted with 10 cohort members who met full ‘functional recovery criteria’ (Clinically Recovered Group) and 10 who did not (Not Clinically Recovered Group). A thematic analysis was performed to develop shared themes and group-specific sub-themes to capture agreement and divergence between groups.

**Results:**

Five shared themes were produced: pursuing balance in conflict, generating meaning in life, experiencing a dynamic personal relationship with time, redressing inequality while managing added challenges/vulnerability, and directing life from resilience to flourishing. The five group-specific sub-themes developed illuminate differences in the meaning ascribed to personal recovery by each group.

**Conclusion:**

Findings emphasise the role of time in how personal recovery is conceptualised by service users and identify ways clinical recovery may influence personal recovery meaning in FEP at mid-later life. Mental health services failing to consider temporal changes in meaning-making and discounting clinical recovery risk ignoring key factors affecting personal recovery.

**Supplementary Information:**

The online version contains supplementary material available at 10.1007/s00127-021-02121-w.

## Introduction

While recovery is a complex, nuanced, and contested construct [1], it nonetheless underpins the ‘recovery approach’, a keystone of modern mental health policy in the global north [2]. Some service users do not identify with or relate to the word; rejecting it as a label reflecting their experiences, ideas, values, and culture [3]; refusing to allow a dominating meaning, explanation, or philosophy to be imposed on them [4]. Despite this, the construct has value. For many, recovery is their preferred term to describe the continuing experience of living with, managing, or overcoming mental health difficulties [5].

There are insufficient data available on how people diagnosed with different forms of psychotic illness conceptualise recovery in mid-later life [6]—this is problematic as understanding of one’s own recovery can change over time [7]. Evidence suggests there are unique aspects of mid-later life recovery, including: perceiving time is running out to improve functioning [8] and having increased capacity to manage and understand psychosis [9]. Long-term data can support mental health services (MHS) to organise themselves according to recovery-oriented frameworks and set objectives when designing models of healthcare provision for older adults. While people can receive multiple diagnoses over time, dissimilar expressions of psychosis impact outcome differently [10, 11]. Therefore, baseline psychotic illness type will likely influence how recovery is understood decades later. To our knowledge, no previous research has investigated how FEP service users (sampled across psychotic illness type) perceive recovery beyond 5 years after diagnosis.

Within the literature, a distinction has been made between personal and clinical recovery [12, 13]. Clinical recovery, defined as remission and social/occupational functioning, is an objective, observable, clearly operationalised, clinician rated, dichotomous construct; its boundaries invariant across persons. In contrast, personal recovery is a multifaceted, individually demarcated, discretely experienced concept incorporating: hope; optimism; identity separate from mental illness; empowerment; meeting responsibilities; agency; self-determination; citizenship; meaning in mental illness experience and life; and connectedness, social integration, and inclusion [14–16].

According to Slade [17], clinical recovery is subordinate to, and a subset of, personal recovery and not a prerequisite to personal recovery progression. A recent meta-analysis concluded that personal recovery is a substantively dissimilar construct to clinical recovery, whose variance is only partially explained by clinical recovery [18]. While quantitative studies are useful in determining the boundaries of recovery taxonomies, qualitative methods explicitly focus on meaning [19]. Therefore, adopting a qualitative approach may be more appropriate to elicit and unravel the congruence and dissimilarity between the two concepts. While there is acceptance that both intersect, it is unknown if recovery meaning (i.e. personal recovery) is affected by clinical recovery status. As far as we are aware, no previous qualitative study has investigated this relationship.

To address these gaps in the literature, we aimed to explore personal recovery meaning with individuals 20 years after their FEP and examine the potential influence of clinical recovery status on how they defined recovery (i.e. personal recovery).

## Methods

### Design, recruitment, and sample

This paper reports on the qualitative aspect of the iHOPE-20 (Irish Health Outcomes in Psychosis Evaluation—20-year follow-up) study. This is a prospective 20-year FEP follow-up study conducted between 2014 and 2017 in Dublin, Ireland. The extent of service user involvement in the study is presented in Table 1. Ethics approval was obtained from the Saint John of God Hospitaller Ministries Research Ethics Committee.

**Table 1:** Extent of service user involvement in study conceptualisation, design, data interpretation, and dissemination

Type of activity	Impact
Appointed to the study steering committee	Safeguarded service user involvement in decision making
Shaped study aims and helped decide on its methodology	Increased the likelihood that the study was grounded in, and relevant to, service users’ lives and helped identify lines of inquiry not previously considered
Co-developed user friendly documentation, helped select assessment instruments, and design the interview protocol	Ensured documentation was accessible and instruments and protocols used reflected service user priorities, experience, and preferences
Enabled the interpretation of findings from non-clinical/academic perspectives	Identified novel insights from the dataset
Contributed to knowledge transfer and exchange activities	Ensured findings were communicated in an effective way, beyond clinical and research communities, to service user and general populations

Study participants were members of an epidemiologically representative FEP incidence cohort of 171 people diagnosed with a FEP between 1995 and 1999 using the SCID-IV (Structured clinical interview for DSM-IV axis I disorders; [20]). We purposefully sampled cohort members who had completed a 20-year follow-up quantitative assessment of outcome (*n *= 80/171). Comparisons between baseline characteristics of these 80 cohort members and those not assessed/deceased at 20 years (*n *= 91) found no statistically significant differences [11]. All 80 potential participants were asked if they were agreeable to contact for the study’s qualitative component. Of these 80, 1 refused and 79 agreed to further contact. Of these 79, 24 were invited to take part and 4 of the invited 24 refused; with 20 agreeing and providing informed consent.

To select the 24 potential participants, we utilised a sampling matrix to pursue maximum variation across the variables: age, sex (male or female), type of psychotic illness diagnosed at baseline (affective or non-affective), and clinical recovery status—defined as ‘full functional recovery’ [21] (Clinically Recovered or not). Full functional recovery is delineated in Table 2. We did not include immigration status or socioeconomic category in our sampling matrix as these data were not available. This, in combination with the absence of race or ethnicity variation among potential participants, meant we were unable to sample for diversity on the basis of social and structural determinants of recovery and disability.

**Table 2:** Definition of full functional recovery

Criterion	Definition
Full functional recovery	A combination of remission of positive and negative symptoms and functional and vocational status recovery
Remission of positive and negative symptoms	Discounting the 6-month duration element, the remission criteria advocated by Andreasen and colleagues [22] was used. A score of ≤ 3 on eight Positive and Negative Syndrome Scale questions [23]: delusions; unusual thought content; hallucinatory behaviour; conceptual disorganization; mannerisms/posturing; blunted affect; social withdrawal; and lack of spontaneity
Functional and vocational status recovery	A score of ≥ 4 on four Quality of Life Scale items [24]: appropriate interpersonal relationships with people outside of family; adequate vocational functioning (paid employment attainment, school participation, homemaker role provision); adequate achievement in role adopted; and basic living task engagement

Two groups were sampled: 10 people who met full functional recovery criteria (Clinically Recovered Group) and 10 who did not (Not Clinically Recovered Group). We were unable to examine the influence of clinical recovery degree as we sampled across clinical recovery status—a binary variable. This is recognised as a study limitation due to the heterogeneity in levels of psychosis symptoms and functioning among the Not Clinically Recovered Group.

In line with the guidance offered by Braun and Clarke [25], an appraisal of ‘information power’ determined when recruitment stopped [26]. This assessment considers study characteristics that influence the dataset quality necessary to achieve objectives. Our study aims were narrow, our sample: highly specific, interview dialogue: mostly strong, the analysis: cross-case, and data interpretation: informed by a theoretical background. We concluded interviewing when information power was deemed sufficient. This was achieved with 20 participants.

### Data collection

Twenty semi-structured interviews, lasting 22–90 min, were conducted. All were guided by an interview protocol (Supplement 1) and centred on eliciting the meaning of recovery to participants (i.e. personal recovery) in the context of a retrospective reflection on their initial FEP and any subsequent mental health difficulties experienced. Questions focused on participants’ perceptions of the term recovery; the images, colours, feelings, and other words they associated with it; and their perspectives on how they classified themselves in recovery. DOK performed 18 interviews; AS completed 2. Interviewers were proficient qualitative researchers unknown to service users before participation.

### Data analysis

All interviews were audio-recorded and transcribed. A thematic analysis was performed using the approach described by Braun and Clarke [27]. Analysis was exploratory, inductive, and essentialist; underpinned by relativism. We aimed to factually report on the experiences, perceived meanings, and reality of participants by analysing data at a semantic level. Data were analysed using NVivo 11 [28] by coding for central ideas, concepts, and patterns which were then assessed for similarities/differences and combined into themes. A set of themes were developed from codes which described participants’ perceptions of personal recovery. To capture agreement and divergence between groups, shared themes and group-specific sub-themes were generated. The factors and forces (behind and beyond clinical recovery status) influencing potential group differences were not examined as these were outside the scope of our study’s aims. Actions taken to address issues of study rigour and reflexivity are outlined in Table 3.

**Table 3:** Actions taken to ensure study rigour and engage in reflexivity

Aspect of the study	Actions taken
Sampling	Participants were selected based on their ability to provide data to enable achievement of the study’s aims
	Justification for sample size and sampling strategy was provided
Data collection	Interviewers had the necessary interviewing skills to listen assiduously, negotiate meaning when aspects of narratives appeared unclear, and respond to participants in a manner that deepened the exploration of the essence of their words
	Interviewers were sensitive to, and tried to be aware of, all participants’ verbal, nonverbal, and non-behavioural communication
Data analysis	Two research team members (DOK and AS) analysed data independently and compared and agreed codes and themes
	Data were interpreted rather than just paraphrased or described
	Thorough engagement with the data ensured themes developed were internally coherent, consistent, and distinctive
Report writing	Assumptions about, and our specific approach to, thematic analysis were clearly articulated
	Language and concepts used in study write up were consistent with the epistemological position adopted
	A balance was achieved between presenting interview extracts to illustrate themes and our analytic narrative so interpretations presented could be judged a reasonable representation of participants’ accounts
All study processes	A detailed audit trail of study processes, the research design, and its implementation was created
Reflexivity	Analytical memos, thoughts, and reflections were recorded, reviewed, and shaped our analysis
	We reflected on how our partial and positioned perspectives impacted knowledge produced by considering how our values, beliefs, academic/clinical training, life experiences, and context affected research processes
	We sought to limit the influence of our preconceptions by actively searching for data that challenged initial interpretations

## Results

**Fig. 1:**
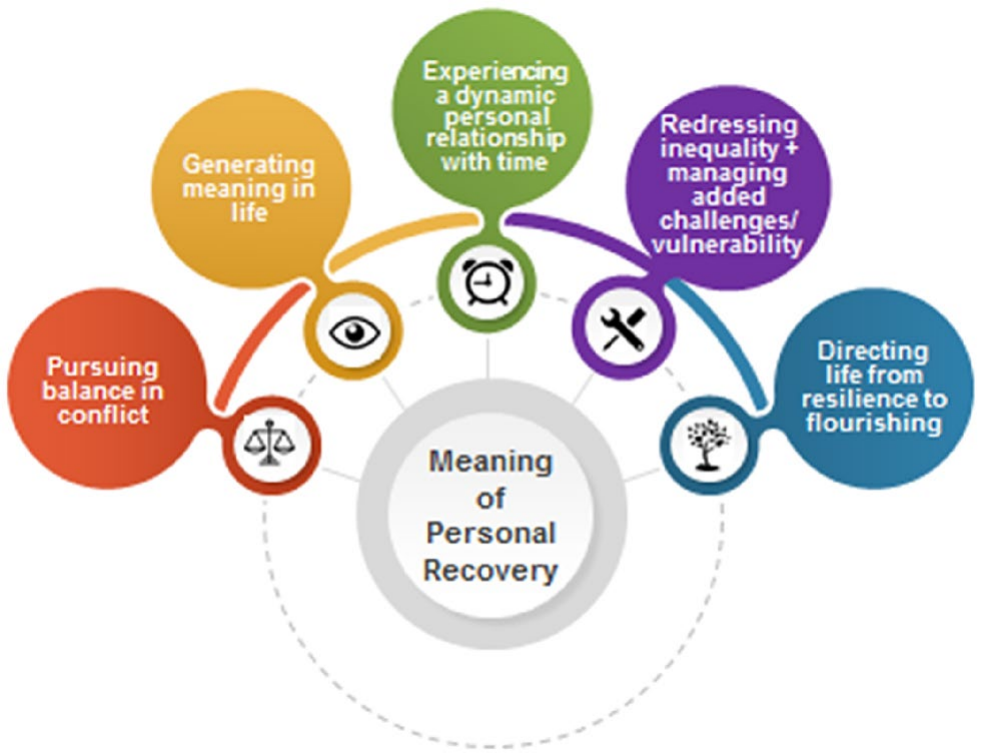
Shared meaning of personal recovery themes

Participant characteristics are presented in Table 4 by clinical recovery status group. The five themes shared by both groups are displayed in Fig. 1 and will now be explained. The five group-specific sub-themes developed are delineated under each corresponding shared theme and illuminate differences in the meaning ascribed to personal recovery by each group. Additional data supporting themes developed are displayed in Supplement 2.

**Table 4:** Demographic characteristics and diagnoses of study sample (*n *= 20)

Characteristic, M(SD)/*n* (%)	Clinically recovered group (*n *= 10)	Not clinically recovered group (*n *= 10)	Entire sample (*n *= 20)
Age in years at time of interview	40.5 (7.26)	46.6 (7.76)	44.55 (7.25)
Race and ethnicity			
White Irish	10 (100%)	10 (100%)	20 (100%)
Gender			
Male	6 (60%)	6 (60%)	12 (60%)
Female	4 (40%)	4 (40%)	8 (40%)
Baseline SCID-IV diagnosis (1995–1999)			
Schizophrenia	3 (30%)	6 (60%)	9 (45%)
Schizophreniform disorder	0 (0%)	1 (10%)	1 (5%)
Delusional disorder	1 (10%)	1 (10%)	2 (10%)
Bipolar disorder with psychotic features	5 (50%)	1 (10%)	6 (30%)
Major depression with psychotic features	1 (10%)	1 (10%)	2 (10%)
Employment status			
Full-time employment	6 (60%)	0 (0%)	6 (30%)
Part-time employment (≤ 30 h per week)	2 (20%)	1 (10%)	4 (20%)
Full-time student (≥ 30 h per week)	1 (10%)	0 (0%)	1 (5%)
Unemployed	0 (0%)	9 (90%)	9 (45%)
Home-maker	1 (10%)	0 (0%)	1 (5%)
Relationship status			
Single	4 (40%)	7 (70%)	11 (55%)
Married	5 (50%)	1 (10%)	6 (30%)
Engaged	0 (0%)	1 (10%)	1 (5%)
Living with partner	1 (10%)	0 (0%)	1 (5%)
Separated/divorced	0 (0%)	1 (10%)	1 (5%)
Highest level of education attained			
Primary level	0 (0%)	1 (10%)	1 (5%)
Secondary level or equivalent	2 (20%)	2 (20%)	4 (20%)
Specific vocational training	0 (0%)	3 (30%)	3 (15%)
Third-level certificate	0 (0%)	1 (10%)	1 (5%)
Third-level diploma/degree	5 (50%)	2 (20%)	7 (35%)
Third-level postgraduate degree	3 (30%)	1 (10%)	4 (20%)

The shared theme *Pursuing balance in conflict* describes both groups’ experience of viewing recovery as living with the dissonance arising from adopting multiple definitions of the concept simultaneously. For participants, recovery meant reconciling these definitions through balance—accepting the contradictions in their interpretations and finding a way to live with ambiguity regarding how they should pursue or engage with recovery. Different apprehensions of the construct were understood by participants to place dissimilar expectations on them (from self and others) leading to the adoption of disparate behaviours.

Ronan frequently experiences homelessness and many members of his family of origin are deceased. In the following extract, he expresses the struggle to reconcile his comprehension of recovery as both a process (living in the moment and accepting illness) and an outcome (having prospects, access to his children, and a romantic relationship):

I live day to day. I don’t plan too much for tomorrow, it [recovery] is really all about the here and the now… you are accepting you have an illness and yet you have the wish to move on… But you [psychosis] have taken everything I have got. You have taken my job, you have taken my opportunity, you have taken my kids, you have taken my ex-girlfriend.

One Not Clinically Recovered Group-specific sub-theme was developed for this theme: *Balancing multiple recoveries*. It describes this group’s experience of prioritising different forms of recovery at different life stages (e.g. recovery from alcohol/substance abuse, childhood trauma, or psychotic experiences themselves) which led to conflict across their life course. For them, recovery meant pursuing balance in this focus over time.

The shared theme *Generating meaning in life* (MIL) describes both groups’ perception of recovery as establishing what made their life meaningful. This involved a reorganisation or reprioritisation of what aspects of life participants wished to invest in following an appraisal of the impact of their FEP and subsequent psychosis episodes on MIL. Participants generated MIL throughout their recovery by: establishing a purpose, a reason for life significance, and life narrative coherence; finding belonging through connectedness with self, others, and nature; becoming socially motivated or sensitive; enacting self-esteem, confidence, and efficacy; and fostering self-knowledge and maturity. Some participants in both groups also viewed recovery as determining the meaning of psychotic experiences and finding benefit in them (e.g. being able to help others in recovery).

Bernadette has significant peer and family support and has been employed for most of her life. From her perspective, connectedness to the sea represents recovery and MIL. Sea swimming helps her re-establish self-connectedness, making her feel like she belongs in her own body and in the natural world, supporting her to care for herself:

[When swimming in the sea] I just felt the feeling of oneness and calmness and being at peace with myself. And I feel that was the start of me looking after myself, physically, and mentally… that to me was the beginning of a feeling I was getting myself back… being ‘at one’ with nature.

Some participants in both groups viewed recovery as a meaningless concept as they did not relate to it. They either felt they had nothing to recover from or believed the term was too ambiguous to be relevant to their lives. Instead, they focused on managing their day-to-day psychological state. Jonah believes his independence largely protects him from psychiatric stigma. This extract from his interview illustrates his rejection of *‘recovery’*:

Recovery is a word I tend to stay away from… I don’t think of myself as recovered or in recovery... I don’t associate with it; it doesn’t have meaning for me… I am just going to live in the moment… be in control of my thoughts, my mental health issues.

One Clinically Recovered Group-specific sub-theme was generated for this theme: *Choosing ‘reality’ over psychosis*. It describes how participants saw recovery as actively deciding to shift their focus and invest their energies away from MIL attained from psychotic experiences toward MIL available to them in consensus reality. Chris attends 3rd-level education, something he attributes to support from MHS and his own agency. In the following extract, Chris describes how, to him, recovery means realising he has more life *‘options’* in *‘reality’* than in the *‘schizo world’*:

I think I am recovered because I am more in tune with what I want out of reality instead of out of the schizo world… Do I want to write about the schizo world and therefore I have to be in the schizo world to write about it or do I want my friends and my family and my college and my future in reality?

The shared theme *Experiencing a dynamic personal relationship with time* describes both groups’ understanding of recovery as their individual connectedness with time changing throughout life. To them, recovery meant living with these changes in temporality. Participants experienced: being stuck in time due to negative life events (e.g. childhood neglect); acquiescing to time (i.e. waiting for life to get better); moving on from one’s past through forgiveness; utilising time to pursue life objectives; and progressing in time by visualising and actualising a positive future.

Elizabeth has extensive social support available to her through a multi-generational family. For her, recovery means being connected to her past, present, and future; yet concentrating on the aspect of her temporality most advantageous to her wellbeing at a particular time:

I just thought, I’m either living in the past and wishing that I didn’t have mental health issues which I had. Or I am looking towards a future that may not be attainable. Or I am just going to live in the moment and just… if I get through each day.

No group-specific sub-themes were produced for this theme.

The shared theme *Redressing inequality while managing added challenges/vulnerability* describes both groups’ interpretation of recovery as reducing the degree to which they perceived others devaluing them following their psychosis diagnosis. This involved participants: meeting family, friendship, occupational, and societal responsibilities; reclaiming power in (and control over) life; being heard, trusted, and respected as an adult human being; relinquishing the *‘sick role’*; and normalising psychosis. Apprehending recovery in this way also involved acknowledging that psychosis brought added challenges (e.g. wanting to avoid people) and vulnerabilities (e.g. loss of control of the self) that differentiated them from others.

Gabriel is unemployed, living in his family home, and regularly attending a rehabilitation day centre. In his interview, he described struggling in his life as an *‘exhausting’* journey to a faraway water source to bring back water with *‘cupped hands’*. From his perspective, people without psychosis *‘walk along the shore’* so they do not need to make this journey. In the following quotation, Gabriel details managing the added challenges that psychosis brings:

From early in my life I am carrying burdens which other people are not carrying… So I have had that experience since the breakdown [Gabriel’s FEP] as well, that in what I do I expend more energy to achieve something which another person will achieve with much less effort.

Two group-specific sub-themes were developed for this theme. Data relating to the first of these (*Repairing my reputation*) were only found in the Clinically Recovered Group. It describes how this group viewed recovery as: accepting, what they considered, past embarrassing or humiliating behaviours linked to psychosis; engaging in career and social network damage control; and regaining social standing. Data relating to the second of these (*Being worthy of investment*) were only found in the Not Clinically Recovered Group. It describes how this group understood recovery as re-establishing self-esteem by perceiving that friends, family, peers, and clinicians valued them and were willing to devote time and energy to understanding and supporting them.

The shared theme *Directing life from resilience to flourishing* describes both groups’ experience of agency in acquiring the determination, personal strength, and inner resources to have the ability to bounce back from setbacks (resilience) and then pursuing wellbeing, happiness, goals/potential achievement, and full engagement with life (flourishing). Developing resilience involved: having structure; learning from mistakes; keeping an open mind; and establishing different identities so if one identity was lost (e.g. employee), there were other identities to fall back on. Fostering flourishing meant: staying grounded; being nurtured by one’s environment; healing the physical and mental self; and cultivating contentment.

Mike is employed and has many sources of instrumental and emotional support in his life. From his perspective, recovery means pursuing flourishing through resilience:

Recovery… I often think of scar tissue or a stronger resilience having gone there and then to come out the other side… having some sort of spark to get up and participate in society… not to opt out or not to be without hope or without any willingness to really engage with day to day life, society, and motivation. Work would be a major part of it… I have since got married, we are lucky enough to have children.

One Clinically Recovered Group-specific sub-theme was produced for this theme: *Breaking through psychosis*. It describes how this group comprehended recovery as interacting with others in a sincere, open, and honest manner (i.e. congruent with their values). This interaction allowed participants to break through and overcome the stifling impact of psychosis on their personality and consequently flourish.

Nuala prioritises sensitivity and empathy in her receipt of MHS. For her, recovery means flourishing by breaking through, what she perceived as, the ‘*ugliness*’ of her psychosis:

I am very kind and I am very loving… and I think that has helped because it takes some ugliness that can exist in the brain out; because you’re nice and kind and lovely. You kinda have to make your own happiness too I think.

## Discussion

### Main findings

The study utilised a unique epidemiological cohort to offer a novel conceptualisation of personal recovery in FEP in mid-later life. An in-depth interpretative account was produced that illuminates entire sample and group-specific meaning-making. Data presented augment understanding of aging service user perspectives and can inform how older people with experience of psychosis can be best supported by MHS. Overall, we found that personal recovery meaning relates to life balance, MIL, temporality, equality, and agency in directing life.

While the primacy of equilibrium in life in personal recovery for older people with schizophrenia has been identified previously [29], our findings suggest one way this balance can be achieved—by holding differing recovery meanings simultaneously and pursuing disparate goals relating to each meaning at different times. They highlight how clinicians should be aware of the definitional conflicts service users may need to reconcile to operationalise ‘recovery’. This suggests mental health policy and services privileging personal recovery as one side of a conflict (e.g. recovery being a process, not an outcome), by applying a one-size-fits-all definition, risk marginalising service users by inappropriately ‘correcting’ their understanding of the concept.

Findings reflect the centrality in personal recovery of meaning-making in psychotic experiences and MIL [30], the tripartite view of MIL (i.e. purpose, significance, and coherence) [31], and the importance of self-belonging and belonging amid others in recovery in psychosis [32, 33]. They also add to this literature by emphasising how belonging can be interpreted as MIL. For participants, belonging was more than just social connectedness; it involved finding their place in the patchwork of existence and connecting to, not just other human beings, but to the self and the natural world.

Findings highlight the potential for the recovery approach’s focus on individualism and personal responsibility to clash with difficulties in experiencing the self as the subject of experience. Psychosis can result in depersonalisation, distortion in first-person perspective, erosion of selfhood coherence/consistency, and disturbances in self-other/self-world boundaries [34]. Personal recovery may be supported by MHS providing interventions designed to enhance self-belonging. For example, Metacognitive Reflection and Insight Therapy explicitly targets the goal of rich and full self-experience by redressing the perception (common in psychosis) that the self is fragmented, lacking coherence, or profoundly different from others [35]. Some participants reported how nurturing nature connectedness enhanced both their sense of belonging amid the natural world and self-belonging. MHS may wish to consider the five pathways to nature connection: contact, emotion, compassion, meaning, and beauty [36]. There may also be a role for supported socialisation to foster social belonging through reconnecting with and extending social networks [37].

Service users reporting that ‘recovery’ is neither meaningful in, nor applicable to, their lives warrant consideration for policy-makers. Policy could promote clinicians communicating, to service users and their supporters, that there are many ways of understanding the experience of living with mental health difficulties (with ‘recovery’ being just one). Examples in our dataset include living in the present, focusing on controlling psychosis/distress, and pursuing health/wellbeing. This could minimise the risk of people not identifying with the construct feeling alienated within, or abandoned by, services.

Our data contrast with how temporality has been understood in mental health recovery previously—personal recovery as reconnecting with time [38]. For participants, personal recovery meant being able to live with changes in temporality over their life course. This finding may be explained by how psychosis can be perceived as changes in the explicit structure of time [39] and a disintegration of basic self-coherence causing disconnection from one’s environment (including time) [40]. MHS considering psychotherapy focused on temporality may help service users live with conflicting temporal perspectives, accept time lost to psychosis, and acknowledge the past, fully experience the present, and hold hope for (and actively shape) the future.

Research exploring early-phase FEP recovery has underscored service users’ desire for equality, societal value, and social inclusion [41, 42]. Our study adds nuance to this knowledge by highlighting how in mid-later life this drive for egalitarianism is balanced against an awareness of the inequity that psychosis brings. Nonetheless, personal recovery for participants meant reclaiming citizenship by being seen by others as responsible, human, and warranting power, trust, and respect in relationships. Citizenship-oriented care can help address structural barriers to citizenship, including poverty, stigma related to employment/housing, and safety issues in the community [43].

Published FEP and schizophrenia data underline the need, in personal recovery, to counteract the perception that one cannot control or effect change in life. This can be achieved by pursuing autonomy and independence to live beyond disability [42, 44, 45]. Findings nuance our awareness of this challenge. They demonstrate how service users can view this ‘regaining of agency’ as wellbeing and full engagement in life actualised by developing resilience as the foundation of flourishing. This resilience groundwork can be strengthened by MHS providing informational, instrumental, and emotional support, while not inadvertently marginalising service users or restricting their social world to mental health contexts [46].

### Impact of clinical recovery

Different forms of discrimination (e.g. psychiatric stigma, racism), interlocking adversities (e.g. poverty, childhood trauma), and macro-structural forces (e.g. discriminatory housing policies, inaccessibility of 3rd-level education) can profoundly impact clinical recovery status [47–49] and thus shape how personal recovery is understood by service users. However, identifying the mechanisms explaining the interrelationship between clinical and personal recovery was not our aim. We conducted an initial exploration of the potential influence of clinical recovery status on personal recovery meaning using a research design epistemologically congruent with clinical recovery (measuring it using standardised instruments) and personal recovery (exploring it in an open ended manner). In doing so, we produced inceptive qualitative findings to help elucidate and clarify this important relationship. We identified considerable agreement and difference between clinical recovery status groups. The group-specific sub-themes generated underscore areas where clinical recovery status may influence personal recovery meaning.

*Choosing ‘reality’ over psychosis* illustrates an agency within the Clinically Recovered Group to actively determine the degree to which they invest in the world of psychosis and allow psychosis to dominate life. This directly challenges the belief that people experiencing psychosis are passive hosts of a brain disorder and is in line with the ability of service users to actively shape and elaborate psychotic experiences [50]. This finding indicates that pursuing clinical recovery may make exercising this agency easier.

For the Clinically Recovered Group, flourishing (as a part of personal recovery) involved *Breaking through psychosis*. This meant using their social abilities to embody their value system to flourish by overcoming psychosis caused barriers to expressing their personality and living an authentic life. The absence of this theme in the Not Clinically Recovered Group suggests that this form of flourishing may be more relevant in personal recovery for clinically recovered service users. The cumulative impact of persistent psychotic experiences on personality may present an added barrier to living authentically [51].

How participants viewed certain challenges and vulnerabilities that psychosis brought was a key site of divergence. The Clinically Recovered Group was concerned with *Repairing my reputation*; the Not Clinically recovered Group with *Being worthy of investment*. This disparity may reflect differences in social and occupational functioning. People with extended social networks adopting a valued social role may be more likely concerned with reputation repair if, when interviewed, they were actively engaged in social systems. Similarly, if a person struggles to find such a role and connect to social systems, they may desire to be seen, valued, and witnessed and thus prioritise others perceiving their worth.

Only the Not Clinically Recovered Group reported *Balancing multiple recoveries*. This may reflect a greater complexity in the recovery journey [52], substance abuse reducing the likelihood of clinical recovery [53], and the association between childhood adversity and psychosis persistence [54]. This group may have more forms of hardship to recover from.

### Limitations and future directions

We did not examine how individual agency and background structural and socioeconomic conditions interact to determine clinical recovery status and shape personal recovery meaning. There was no ethnic or racial variation among the sample; most participants were male, single, and had obtained a third-level qualification. People who had a pre-established interest in (or affinity with) the concept of ‘recovery’ may have been more likely to take part. Group differences found may be a reflection of our sample (e.g. spread of psychotic illness type) or other unmeasured factors rather than the influence of clinical recovery status. The restricted extent of service user involvement in the study is also a limitation.

By examining the agency–structure nexus, critical realist and the capabilities approach informed research could help illuminate the role of structural configurations that generate inequality, impede clinical recovery, and limit the embodied experience of the aspects of personal recovery we identified. Such research may further clarify the clinical-personal recovery relationship, help identify and remove oppressive structures, and guide ameliorative social change [55, 56]. As clinical recovery’s influence may differ depending when meaning-making occurs, studies exploring the relationship at different time points post psychiatric diagnosis would be helpful. To investigate the influence of clinical recovery degree on personal recovery meaning, research using multiple clinical recovery categories is warranted. Further efforts to address why (outside of clinical recovery’s influence) personal recovery is conceptualised in particular ways would be of value. Future studies should consider sampling across immigration status, socioeconomic category, race, and ethnicity. Finally, service user led research and augmented service user involvement may generate novel insights.

### Conclusion

Findings emphasise the role of time in how personal recovery is conceptualised by service users and identify ways clinical recovery may influence personal recovery meaning in FEP at mid-later life. MHS failing to consider temporal changes in meaning-making and discounting clinical recovery risk ignoring key factors affecting personal recovery.

## Supplementary Information

Below is the link to the electronic supplementary material.

Supplementary file1 (DOCX 20 KB)

Supplementary file2 (DOCX 18 KB)

## Data Availability

The study dataset will not be made publicly available because we never obtained ethical approval or consent to share participants’ data in this manner.
