# Supplementary material for: A qualitative study exploring personal recovery meaning and the potential influence of clinical recovery status on this meaning 20 years after a first-episode psychosis
Source: Soc Psychiatry Psychiatr Epidemiol. 2021 Jun 18;57(3):473–83. doi: 10.1007/s00127-021-02121-w (PMC8934321; doi:10.1007/s00127-021-02121-w)
Supplement: Supplementary file 1 — Supplementary file1 (DOCX 20 KB) [file 127_2021_2121_MOESM1_ESM.docx]

Supplement 1 Full interview protocol

**Introducing the interview**

NOTE: Thank participant for agreeing to take part in the study and for giving you their valuable time.

In this interview I am going to ask you about what recovery means to you in the context of your first-episode psychosis and any subsequent mental health difficulties you may have experienced. By ‘psychosis’ I mean: sensing things others cannot sense; believing things others find strange; feeling out of touch with reality; having difficulty feeling emotions, experiencing pleasure, or speaking; or not having motivation to be social or to do the things you want to do*.* By ‘first-episode psychosis’ I mean the time you had first contact with mental health services due to your experience of psychosis approximately 20 years ago.

**Questions**

Thinking about your first-episode psychosis and any subsequent mental health difficulties you may have had…

1. What does the word recovery mean to you?

Prompts: How do you understand recovery? How do you interpret it? How do you make sense of it? Why does recovery mean that to you?

1. Is there an image you would use to describe recovery? Why does that represent recovery to you?

Prompts: Maybe a picture, a person, a place, or an object?

1. Is there a word that you would use to describe recovery? Why do you use that word?

Prompts: What language do you use to describe it?

1. How do you feel about recovery?

Prompts: Does the word make you feel any particular emotions?

1. Do you feel you are recovered? Why do you feel this way?

NOTE: Ensure use of use participants’ language. Prompts: For example, why do you feel you are ‘unsure’, ‘fully’, ‘partially’, or ‘not recovered’ or that ‘recovery is a process’?

1. Is there anything else you would like to add?

**Ending the interview**

NOTE: Thank the participant for sharing their perspective and experiences with you. Reiterate that all data provided will be treated with respect and that safeguards are in place to protect their identity. Ask the participant how they are feeling after the interview.
