# Supplementary material for: A qualitative study exploring personal recovery meaning and the potential influence of clinical recovery status on this meaning 20 years after a first-episode psychosis
Source: Soc Psychiatry Psychiatr Epidemiol. 2021 Jun 18;57(3):473–83. doi: 10.1007/s00127-021-02121-w (PMC8934321; doi:10.1007/s00127-021-02121-w)
Supplement: Supplementary file 2 — Supplementary file2 (DOCX 18 KB) [file 127_2021_2121_MOESM2_ESM.docx]

Supplement 2 additional interview extracts to support themes presented

| **Theme** | **Sub-theme** | **Interview extract** |
| --- | --- | --- |
| Pursuing balance in conflict. |  | ‘Recovered?’ is a hard question to answer because it’s a continuous illness. There’s no cure for it. So it depends on what you want to classify as recovered. Recovered from a psychosis? Yes. But recovered to the state of leading a life without the illness? No. The closest you could feel to recovery I suppose is contentment and happiness. |
|  | Balancing multiple recoveries | Recovery, well… getting over what happened to me when I was younger [exposure to trauma]. That would have caused one breakdown anyway. And then I would have had another breakdown over taking drugs because I messed up my mind because of drugs, and then the alcohol situation, that wasn’t great either. |
| Generating meaning in life. |  | I have a purpose to get up in the morning and go out, maybe do a garden maybe. I have sisters and brothers, and I am the only one that is good with my hands, I would be helping them with stuff, fixing stuff in their houses, doing their garden and things like that. So that keeps me going as well, it is not just for the money. What I would get out of it would be good. |
| Experiencing a dynamic personal relationship with time. |  | [Recovery means] going to college. The accomplishment I suppose, being given tasks to do so you would be kept busy, you are not solely thoughtful of your own thoughts. If I didn’t do it, I’d say I would be still stuck in the same place, losing time. Now I’m moving forward. |
| Redressing inequality while managing added challenges/ vulnerability. |  | It [mental health] is something that I always have to be very mindful of, that it can take me rather than I take it, so in ways I would always deeply respect it, if that makes sense, and not kind of push it too much… You are the same as everybody else, you might have to mind yourself a bit more and sleep a bit more and things like that. But I never used it as an excuse. I would have liked to, it could get you out of jail sometimes if you wanted it to, you could easily say: ‘No I can't do this, I am not able’. But sure then you’d feel bad and then you’d feel weak. |
|  | Repairing my reputation | So it was very much about accepting [having experienced psychosis at work] and then the damage control after that experience, because that would have been something that would have been seen as quite personal. Your diagnosis was then known and you were trying to manage that and deal with that. So that was a challenge as there was an extra chapter on top of recovering from the relapse. Then you are going back to work and then you are dealing with the fallout from that. |
|  | Being worthy of investment | I think about it...not being able to repay them [clinicians] the way I’d like to but then their attitude is: ‘Well this is our job. If we see you leaving here, we have succeeded. We know that we have succeeded in something, we can go out and have a few drinks and be able to say, right she's finished now, I wonder what's going to happen to her? You know, for the future’. That to me was lovely… that I deserved that. |
| Directing life from resilience to flourishing. |  | I was having problems with my life, sorting them out but only in a very sort of restricted fashion, bouncing back, but problems were all the time developing. Recovery then is sort of like the second half of my life… Living the best life I can, having insight so that I am able to sort of cope with what happens in my life. I suppose the image would be the image of caterpillar-butterfly. The caterpillar doesn't fly, the butterfly flies. |
|  | Breaking through psychosis | It [recovery] means being able to interact with people in a normal basis, being able to hold down a job, being able to deal with people in a matter that kind of fits my personality. My illness affected my personality. |
